# IMPROVING THE ENGAGEMENT OF RESIDENTS WITH DEMENTIA IN ADVANCE CARE PLANNING DISCUSSIONS IN LONG-TERM CARE

**DOI:** 10.1093/geroni/igad104.1781

**Published:** 2023-12-21

**Authors:** Sharon Kaasalainen, Abigail Wickson-Griffiths, Shirin Vellani, Tamara Sussman

**Affiliations:** McMaster University, Hamilton, Ontario, Canada; University Of Regina, Regina, Saskatchewan, Canada; McMaster University, Hamilton, Ontario, Canada; McGill University, Montreal, Quebec, Canada

## Abstract

As part of the Strengthening a Palliative Approach in Long Term Care (SPA-LTC), we have designed and evaluated a number of tools and resources to promote a palliative approach in dementia, including a Canadian version of the Conversation Starter Kit (CSK) booklet. We used participatory action research to design tools for use with care providers based on survey data and interviews. Data was analyzed using thematic analysis and descriptive statistics. We found that residents reported that they engaged in asking more questions to their care provider after completing the CSK booklet. However, care providers stated that they did not feel comfortable addressing questions and concerns raised by residents and their family members. Hence, we co-designed two tools to help build capacity among care providers to support ACP discussions with residents and families, including an e-learning module about ACP discussions for people with dementia and a Conversation Guide. Pilot findings of these tools suggest that staff appreciate the information and tools to enable them to initiate ACP discussions and address difficult conversations. Clearly, residents with dementia and their families need to be supported to engage in ACP discussions early on in their disease trajectory so that residents are afforded the opportunity to voice their values and wishes for end of life care. At the same time, care providers need to have the skills (e.g., listening, probing) and comfort to engage in these discussions to support and help prepare residents and families for important decisions that need to be made later on.

